# Verruca plana on a tattoo: A case report

**DOI:** 10.1097/MD.0000000000019744

**Published:** 2020-04-10

**Authors:** Yan Jing Chen, Owais Nabi, Ping Diao, Ruo Yu Wan, Li LI

**Affiliations:** West China Hospital, Sichuan University, China.

**Keywords:** tattoo, verruca plana

## Abstract

**Rationale:**

Tattooing is a procedure where ink is inserted typically in the intraepidermal space of the skin. Multiple incidences of viral infections following tattooing which lead to warts have been reported in recent years. The aim of this report was to show a relatively rare adverse effect after tattooing – verruca plana.

**Patient concerns:**

A 27-year-old female presented to our department with complains of multiple verrucous papules over her 2-year-old tattoo without itch.

**Diagnoses:**

Pathological investigation confirmed the diagnosis as verruca plana.

**Interventions:**

The patient was treated with 3 cycles of liquid nitrogen cryotherapy and 5% imiquimod cream for 5 months.

**Outcomes:**

A significant improvement in her lesions was observed after the combined treatment.

**Lessons:**

Clinically, verruca plana post-tattooing is relatively less reported. We need to combine clinical manifestations with pathological results to arrive at a definitive diagnosis. Besides, there are a large numbers of post-tattoo complications and various routes of virus inoculation. Therefore, it is important for medical professionals to caution people before considering to have a tattoo.

## 1 Introduction

Tattooing is a procedure where ink is inserted typically in the intraepidermal space of the skin. Recently, having a tattoo has become increasingly popular for it's artistic, beautifying and blemish-concealing features. However, in many cases the adverse reactions post-tattooing produce undesirable outcomes. The main side effects caused by tattooing include immunological, inflammatory, infectious, and neoplastic complications.^1^,^2^,^3^ Viral infections along with bacterial, fungal, parasitic infections are dominant components of infectious complications.^[2]^ Currently there are only a few reports about warts that developed after tattooing.^4^,^5^,^6^,^7^,^8^,^9^,^10^ And rare human papillomavirus types had been detected among the reported literatures. As far as we know, human papillomavirus (HPV) 6B, 27, 47 can be seen in tattoo-associated HPV-induced verruca.^6^,^7^ Herein, we report a relatively rare case of tattoo-associated verruca plana.

## 2 Case report

**Figure 1:**
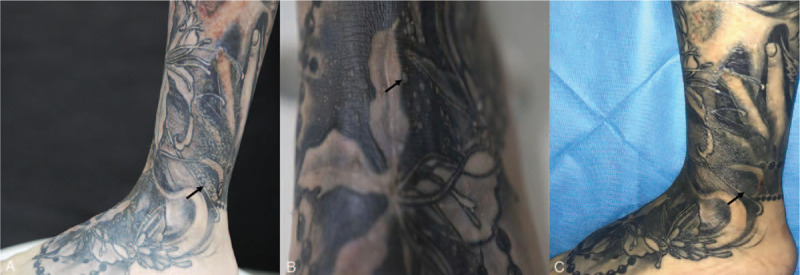
Main clinical manifestations: (black arrows) papules on the lower left leg (A) and dorsum of foot (B). Papules disappear after treatments (C).

**Figure 2:**
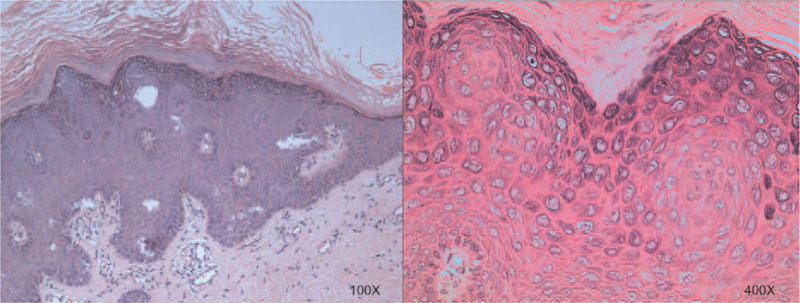
Hyperkeratosis of epidermis, hypergranulosis and acanthosis, koilocytes in the granular layer; (Hematoxylin-eosin stain; original magnification; ×100/400).

A 27-year-old female presented to our department with complains of multiple verrucous papules over her 2-year-old tattoo without itching. The tattooing had been performed to conceal a scalded scar on the left leg present for more than 1 year. At first the papules appeared above the tattooed area, eventually involving the lesions with larger numbers. On physical examination, she had 1- to 4-mm size of verrucous papules on her left dorsal foot and the anterolateral side of the left lower leg within and limited to her black tattoo (Fig. 1A and B). No analogous lesions were observed anywhere on the rest of her body. No past-history of warts could be established. We analyzed the tattoo picture by Photoshop CC 2019 and we found the color of the tattoo varied from the light grey to black, including dark-blue. The average R = red, G = green, B = blue (RGB) values are 50, 53, 56 separately. After checking with the tattoo artist the chemicals in the dye were confirmed to be iron oxide, carbon, magnetite, and so on. Histopathological examination found hyperkeratosis of epidermis, hypergranulosis, and acanthosis. A significant proliferation of koilocytes could be seen in the granular layer (Fig. 2). Finally, the definitive diagnosis of this case was made as verruca plana. The patient was treated with 3 cycles of liquid nitrogen cryotherapy and 5% imiquimod cream for 5 months. A single cryotherapy cycle was given once a month and each one lasted for 3 to 5 seconds only (twice freeze and thaw cycles per lesion). Marked improvement in her lesions was observed after the combined treatment. As pointed by the black arrow, several bulged papules and plaques above the tattoo have almost disappeared. The lesional surface has also flattened (Fig. 1C). No recurrence was observed in a 5-month follow-up period.

## 3 Discussion

Tattoos have become increasingly popular among a large section of population especially younger people. Among the most preferred colors are dark, red, and multicolor. Post-tattoo complications differed according to various colors used in tattooing. For example, “papulonodular” pattern often develops above dark tattoos associated with nonallergic reactions while red tattoos always manifest as “plaque elevations” or plaque-like patterns, “excessive hyperkeratosis”, “ulceronecrotic” patterns which indicates allergic reactions.^[1]^

Viral infection is one of the most common delayed complications after tattooing for HPV inoculated warts can have a long latency period ranging from 2 months to 10 years.^7^,^9^ It involves systemic as well as localized infections. Hepatitis B and C, human immune-deficiency virus have relatively higher risk of systemic inoculation and subsequent infection during tattooing. Warts, Condylomas, and Herpes Simplex are often seen in localized cutaneous infection.^1^,^2^,^3^ There are several case reports about verruca vulgaris on tattoo in the literature.^4^,^5^,^7^ Most cases involved lesions mainly localized on the black or dark-blue dye areas. However, neither verruca plana nor its predilection colors have been widely reported.

Previous reports have mentioned the routes of virus transmission and supported that HPV can be traumatically inoculated^[9]^ and inclined to appear above the black or dark-blue colours.^7^,^10^ In general, it proposed that the HPV could be inoculated through contaminated instruments, ink, artist's saliva, or preexisting but unnoticed warts in the tattooed area.^3^,^9^,^10^ In our patient, there was no history of previous warts. Otherwise, no long-existing trauma has been identified as a risk factor which link to any tattoo-associated adverse effects. Therefore, neither the particular colour pigment nor fresh trauma from multiple needle piercings^[3]^ could be excluded as the risk factors of the inoculation. Factors like contaminated instruments and artists’ saliva could not be ruled out either.

Verucca plana could be diagnosed by experienced dermatologists with the help of its classical clinical manifestations and histopathologic examinations. However, to identify the presence of the virus infection and to confirm the subtype of HPV could provide more useful information for untrained dermatologists who need ascertained evidence of the diagnosis. With analysis of an immunohistochemical result, PCR and DNA sequencing of the virus, Krecke et al^[6]^ found positive nuclear stain for HPV in the stratum corneum of the corneocytes and proved the presence of cutaneous β1-HPV type 47 DNA. Regretfully, tests of PCR, etc, which have been mentioned above were not performed in our case.

The treatment modalities for verruca plana or vulgaris include cryotherapy, curettage, photodynamic therapy, podophyllotoxin, topical imiquimod (5%), or Veregen 10% ointment, which lead to different outcomes.^4^,^6^,^7^,^9^ Due to the optimal standard treatment method and minimum adverse effect, the patient was treated with liquid nitrogen cryotherapy. As for the response to the intervention, unlike the previous cases,^4^,^9^ our patient's skin lesions resolved after concomitant treatments with 3 cycles of cryotherapy and 5% imiquimod cream. The treatment did not leave behind any pigmentation. Imiquimod cream was applied to the skin lesions 1 week after cryotherapy. Fortunately, the patient responded well to the combined treatment. And we did not notice any recurrence or spread on the tattoo or perilesional area within the next 5-month follow-up period.

## 4 Conclusion

To sum up, this case report presents a relatively rare virus-associated side effect of tattooing. Tattooing is considerably risky although it is beautifying. As a doctor, we have the obligation of warning people to be tattooed only in certified institutions. And immediate medical advice should be sought from dermatology professionals in case of a reaction or an undesirable condition post-tattooing.

## Author contributions

**Supervision:** Li LI.

**Writing – original draft:** Yanjing Chen.

**Writing – review & editing:** Yanjing Chen, Owais Nabi, Ping Diao, Ruoyu Wan.
